# Structural Basis for Substrate Specificity in Human Monomeric Carbonyl Reductases

**DOI:** 10.1371/journal.pone.0007113

**Published:** 2009-10-20

**Authors:** Ewa S. Pilka, Frank H. Niesen, Wen Hwa Lee, Yasser El-Hawari, James E. Dunford, Grazyna Kochan, Vladimir Wsol, Hans-Joerg Martin, Edmund Maser, Udo Oppermann

**Affiliations:** 1 Structural Genomics Consortium, University of Oxford, Headington, United Kingdom; 2 University of Kiel, Kiel, Germany; 3 Faculty of Pharmacy, Charles University, Hradec Kralove, Czech Republic; 4 Nuffield Department of Orthopedic Surgery, Rheumatology and Musculoskeletal Sciences, Botnar Research Center, Biomedical Research Unit, University of Oxford, Oxford, United Kingdom; University of Cambridge, United Kingdom

## Abstract

Carbonyl reduction constitutes a phase I reaction for many xenobiotics and is carried out in mammals mainly by members of two protein families, namely aldo-keto reductases and short-chain dehydrogenases/reductases. In addition to their capacity to reduce xenobiotics, several of the enzymes act on endogenous compounds such as steroids or eicosanoids. One of the major carbonyl reducing enzymes found in humans is carbonyl reductase 1 (CBR1) with a very broad substrate spectrum. A paralog, carbonyl reductase 3 (CBR3) has about 70% sequence identity and has not been sufficiently characterized to date. Screening of a focused xenobiotic compound library revealed that CBR3 has narrower substrate specificity and acts on several orthoquinones, as well as isatin or the anticancer drug oracin. To further investigate structure-activity relationships between these enzymes we crystallized CBR3, performed substrate docking, site-directed mutagenesis and compared its kinetic features to CBR1. Despite high sequence similarities, the active sites differ in shape and surface properties. The data reveal that the differences in substrate specificity are largely due to a short segment of a substrate binding loop comprising critical residues Trp229/Pro230, Ala235/Asp236 as well as part of the active site formed by Met141/Gln142 in CBR1 and CBR3, respectively. The data suggest a minor role in xenobiotic metabolism for CBR3.

**Enhanced version:**

**This article can also be viewed as an enhanced version in which the text of the article is integrated with interactive 3D representations and animated transitions. Please note that a web plugin is required to access this enhanced functionality. Instructions for the installation and use of the web plugin are available in Text S1.**

## Introduction

Reduction of carbonyl groups to the corresponding alcohols constitutes a significant metabolic step both for endogenous and xenobiotic compounds [1]–[3]. These reactions are carried out by distinct NAD(P)(H) dependent oxidoreductases mainly belonging to three protein superfamilies, namely the short-chain dehydrogenases/reductases (SDR), aldo-keto-reductases (AKR), or medium-chain dehydrogenases/reductases (MDR) [1]–[3].

A unifying feature of carbonyl reductases appears to be their broad and often overlapping substrate specificity. Endogenous substrates comprise steroids, eicosanoids, cofactors, neurotransmitters and polyols. In addition, a large set of xenobiotics has been identified as substrates for carbonyl reducing enzymes including drugs such as warfarin, daunorubicin or ketoprofen, environmental pollutants (PAH quinones derived from diesel exhaust such as phenanthrenequinone) or tobacco derived carcinogens like NNK [2], [3].

In humans, several enzymes contribute significantly to the metabolic reductive transformation, mainly found in tissues such as liver, kidney, placenta or the central nervous system. The major cytosolic enzymes identified are the NADPH-dependent carbonyl reductase (CBR1, according to the official nomenclature system SDR21C1)[4], [5], belonging to the SDR family, and members of the AKR family such as aldehyde reductase (AKR1A1), aldose reductase (AKR1B1), several dihydrodiol/hydroxysteroid dehydrogenases (of the AKR1C subfamily) or aflatoxin aldehyde reductase (AKR7A2) [1]–[3], [6].

CBR1 fulfills an important role in the phase I metabolism of xenobiotics. Substrates include o-quinones derived from polycyclic aromatic hydrocarbons (PAH) or p-quinones such as menadione, besides an extraordinarily wide spectrum of xenobiotic carbonyls such as anthracyclines, metyrapone or the carcinogen 4-methylnitrosamino-1-(3-pyridyl)-1-butanone. The endogenous compound spectrum comprises steroids, eicosanoids and lipid derived aldehydes [7]–[9].

Recent studies indicate a role for CBR1 in apoptosis, tumor metastasis and oxygen induced stress [10]–[12]. At present, no clear evidence is available which specific endogenous substrate is responsible for these effects, however a recent study showed that CBR1 effectively inactivates in vitro the lipid aldehyde 4-oxononenal [13], indicating that CBR1 is involved in the stress response and elimination of metabolites produced by reactive oxygen species.

A human paralog, *CBR3* (SDR21C2)[4], [5], which is 71% identical to CBR1 [6] is located in the vicinity of the *CBR1* gene on chromosome 21 at 21q22.12. However, thus far limited information on enzymatic properties of CBR3 is available [14]; in this and another study [15] only CBR3 activity towards the model substrate menadione, 4-nitrobenzaldehyde or 4-benzoylpyridine was reported. Given the importance of CBR1 and other carbonyl reductases in endogenous and xenobiotic carbonyl metabolism, we performed a study to establish a substrate specificity profile of human CBR3. To understand structure-function relationships between the two related human CBR enzymes, we analyzed substrate specificity features of CBR1 and CBR3, and furthermore establish a structural basis for the activity differences through mutational, kinetic and crystallographic studies.

## Materials and Methods

### Cloning and Mutagenesis

A human CBR3 clone was obtained from the MGC clone collection, and a synthetic, codon-adapted CBR1 clone was obtained from GenScript Corporation. CBR1 and CBR3 expression constructs were cloned by PCR into pNIC28-Bsa4 or p11-Bsa4, which are T7/pET21a derived expression vectors containing Tobacco Etch Virus (TEV) protease cleavable N-terminal hexahistidine tags. All CBR1 and CBR3 mutants were generated from the vector template encoding the wild type (WT) gene by using a site-directed mutagenesis kit (Quick change, Stratagene). Sequences of all wild-type and mutant constructs were verified by DNA sequencing.

### Expression and purification of CBR proteins

Expression plasmids were transformed into competent Rosetta *E. coli* cells. Protein expression was induced at 18°C at an OD_600_ = 1 by adding isopropyl-1-thio-β-D-galactopyranoside to a final concentration of 0.5 mM to cultures grown in Terrific Broth, supplemented with 50 µg/ml kanamycin or 100 µg/ml ampicillin at 37°C. Induced cultures were then incubated overnight with shaking at 18°C. Cell pellets were resuspended in 50 mM HEPES pH 7.5, 500 mM NaCl, 5 mM imidazole, 5% glycerol and protease inhibitors (EDTA-free Complete, Sigma). Cells were lysed using a high pressure homogenizer (EmulsiFlex-C5, Avestin), followed by centrifugation at 37,000 x g for 45 min.

The supernatant was loaded on an AKTA-Express system (GE/Amersham) and purified using nickel-affinity chromatography on a HisTrap FF 1 ml column (GE/Amersham) and gel filtration on a Superdex 200 column (GE/Amersham) equilibrated in 10 mM HEPES pH 7.5, 500 mM NaCl, 5% glycerol, 0.5 mM TCEP.

For crystallization purposes, CBR3 wild-type fractions from gel filtration were subjected to TEV cleavage overnight at 4°C and the cleaved protein was purified on IMAC-Sepharose (GE/Amersham). The final step of this purification was ion-exchange chromatography on a QHP column (GE/Amersham) using a 0.05–2 M NaCl gradient in 50 mM HEPES pH 7.5, 0.5 mM TCEP, followed by a subsequent buffer-exchange into gel filtration buffer (as above). All purification steps were analyzed by SDS-PAGE and the molecular weight of purified protein was verified by electrospray mass ionization-time-of flight mass spectrometry (Agilent LC/MSD time-of-flight). Proteins were concentrated to 5–10 mg/ml in an Amicon Ultra-15 concentrator with a 10 kDa mass cut-off and the final concentration was measured by UV-spectroscopy (Labtech, Nanodrop 1000 spectrophotometer). Proteins were flash-frozen in liquid nitrogen until further use.

### Substrate screening of CBR1 and CBR3 proteins

Frozen aliquots of CBR1 and CBR3 enzymes were thawed quickly in water of RT and immediately placed on ice. Assays were performed at 30°C in buffer S (50 mM sodium phosphate, pH 6.8, 150 mM NaCl, 1 mM MgCl_2_, 2% (v/v) DMSO). The final assay solution contained 200 nM of protein, 200 µM NADPH and 200 µM of compound. Prior to the start of the experiment, each protein was incubated in buffer containing 1 mM NADPH at 1 µM enzyme concentration for 10 minutes at RT. A solution comprising NADPH in buffer alone was used for the setup of control experiments for each compound tested. Dilutions of compounds at 10 mM concentrations in DMSO were prepared in 96-well plates and used to set up the assay plate (384-well white PCR plate, Bio-rad), by adding 200 nl of each solution into 7.8 µl of buffer S (STARlet nano, Hamilton). The assay plate was centrifuged (1 min, 1,000 rpm, RT) to collect all solutions in the bottom of the wells. Reactions were performed on 24 wells at a time in a filter-based fluorescence reader (Omega Polarstar, BMG Labtech). After one minute of monitoring the fluorescence intensity (excitation, 355 nm; emission, 460 nm) the reactions were started with injections from the instrument-controlled syringe, of 2 µl/well of protein/NADPH solution (see above). The fluorescence intensity in all 24 wells was then monitored for additional 10 minutes. The next set of reactions were afterwards automatically started and measured via the instrument's script mode until all wells of the plate were read. In total the time for the measurement of a complete set of triplicates for 96 conditions was approximately 90 minutes. Data were analyzed for the initial rates of activity, by regression in the linear region of the curves as appropriate. Protein-independent, ‘background’ activities were subtracted and corrected for compound effects, e.g. quenching, by normalization to the fluorescence offset that resulted from the injection of NADPH. Specific activities (in µmol/min/mg) were calculated using the molecular weight of the protein.

### Kinetic analysis of CBR proteins

The kinetic measurement for oracin was performed employing a HPLC method (Agilent 1100 Series, Agilent Technologies, Waldbronn, Germany). Samples were incubated for 60 min and reactions were stopped by the addition of 80 µl of 30% ammonia and cooling on ice. The mixtures were extracted twice with 500 µl of ethylacetate and the combined organic phases were evaporated under vacuum. The residue was dissolved in the mobile phase and analyzed by HPLC (mobile phase, 10 mM hexanesulfonic acid and 50 mM triethylamine adjusted to pH 3.3 with H_3_PO_4_; flow, 1.5 ml/min; 5 µM BDS; Hypersil C18 column (4×250 mm, 5 µm, Thermo Electron Corporation, Cheshire, UK)). The fluorescence emitted at 418 nm was monitored upon 340 nm excitation. The increase of the product concentration was linear over the measurement time

Catalytic properties for isatin were determined by measuring the decrease in absorbance at 340 nm (Cary 100 scan photometer, Varian, California, USA). A reaction mixture consisted of substrate, 500 µM NADPH, 100 mM Tris–HCl pH 7.4, and enzyme. The enzyme solution was diluted in the corresponding elution buffer, a 7∶3 mixture of 10∶500 (mM) imidazole buffer, to ensure that substrate consumption was linear over time. Each concentration was measured at least three times.

The reaction temperature was held constant at 25°C. After a preincubation time of 2 min 10 µl of enzyme solution were added to 790 µl of reaction mixture. A reference cuvette contained the reaction solution without enzyme. Isatin stock solution was prepared in DMSO. The final concentration of DMSO in the reaction mixture was 10% (v/v). A maximum of 4000 µM isatin was used in the kinetic measurement as the change in absorbance of this concentration still follows Lambert–Beer's law and no precipitation of isatin occurred. The kinetic constants were calculated by nonlinear regression (Gnuplot 4.2) with a molar extinction coefficient for NADPH of 6.22×103 M−1 cm−1.

For the determination of the kinetic constants for the activity of the enzymes towards 1,2-naphthoquinone and 1,4-naphthoquinone a modified version of the protocol used for substrate screening (see above) was applied: In a 96-well microplate, 12 concentrations of each substrate, covering ranges from 0 to 4 mM or 0 to 7.5 mM of 1,2- and 1,4-naphthoquinone, respectively, were set up in two rows and then dispensed into the remaining rows to fill the entire plate. This was then used as pre-plate for the assay as described above. The resulting assay concentrations of the two substrates spanned, thus, ranges from 0 to 1 mM or from 0 to 1.875 mM, respectively. Linearity in the protein-independent reduction of compound was observed up to the applied maximum concentrations, thus showing that the compounds were soluble up to that concentration. The resulting data were fitted to the Michaelis-Menten equation using non-linear regression (Levenberg-Marquardt) calculated with Gnuplot 4.0 (http://www.gnuplot.info) or with Prism 5.0 (GraphPad Software, Inc.). In cases where a fit was not possible, due to a failure to plateau, a value of k_cat_/*K*_m_ was estimated from a linear regression over the initial part of the curve. Very small activites (compared to background) were regarded noise below a threshold for the goodness of fit (R^2^) of 0.5 (for all other data values of R^2^ were 0.9 or higher)

### Crystallization of human CBR3

Frozen protein was quickly thawed, and 5 mM NADP was added to the protein aliquot prior to crystallization. A sitting drop consisting of 50 nl of protein solution and 100 nl of well solution was equilibrated against a well solution containing 1.8 M tri-ammonium citrate pH 7.0 at 20°C. Large, irregular crystals that appeared after 24 hrs, were cryo-protected in a mixture of well solution with 25% glycerol in the presence of NADP before flash-cooling in liquid nitrogen.

### Data Collection, Phasing and Refinement

The native dataset was collected on a Rigaku FRE-Superbright generator with R-AXIS HTC area detector. Initial phases were calculated by molecular replacement using the crystal structure of human CBR1 (PDB 1wma) as a model for PHASER [16]. Before the refinement commenced, 5% of the data was flagged during processing for the calculation of R_free_. The final model was created by alternating rounds of the refinement using REFMAC5 [17] and model building with adding ligand and solvent molecules using COOT [18]. The final statistics for the CBR3 binary complex structure are given in supplementary information Table S2.

### CBR3 loop modelling

The active site loop of CBR3 was identified and submitted to a search against an ICM built-in library containing suitable loops with matching ends and as close to the sequence as possible. The algorithm then inserts the matched loops into the model and modifies the side-chains according to the model sequence. The next step adjusts the best loops found and keeps a stack of alternatives. We have manually browsed through the alternatives until identifying a suitable conformation that satisfied the condition of being part of the cofactor binding cavity (as seen in CBR1) and not bearing major atom clashes. The suitable loops were then submitted to local minimisations, with the side chains allowed to move along the chi angles in order to solve the remaining clashes. Upon solution of clashes, the modelled loop was accepted and the resulting model was saved.

### Substrate docking

Docking procedures were performed according to the methodology described and implemented in the program ICM v.3.4-9d[19]. Three different protein structures were used in the docking procedure as receptors: human CBR1 (PDB 1wma), human CBR3 (PDB 2hrb) and human CBR3 with the active site loop modelled as a variant of the conformation adopted in human CBR1. Each of the receptors was docked with seven ligands: 1,2-naphthoquinone, isatin, oracin, menadione, metyrapone, oxononenal and NNK. In each docking procedure, grid maps representing different properties of the receptor were computed. During the docking, either one of the torsional angles of the ligand was randomly changed or a pseudo-Brownian move was performed. Each random change was followed by 100 steps of local conjugate-gradient minimization against the grid maps. The new conformation was accepted or rejected according to metropolis criteria using a temperature of 600 K. The length (number of Monte Carlo steps) of the docking run as well as the length of local minimization was determined automatically by an adaptive algorithm, depending on the size and number of flexible torsions in the ligand. Visual inspection was performed for the lowest energy conformations satisfying the absence of clashes after docking.

## Results

### Substrate screening of CBR3 and comparison to CBR1

Human CBR3 was expressed as N-terminally His_6_-tagged protein in *E. coli* and purified to apparent homogeneity by consecutive chromatographic steps comprising immobilized metal affinity and size-exclusion chromatography. The enzyme was subjected to a substrate screening against a focused library of 111 different carbonyl substrates, using spectrophotometric and HPLC-based assays. The library consisted of a variety of endogenous carbonyl containing ligands such as polyols, eicosanoids or steroids, as well as a diverse set of xenobiotic carbonyl compounds, shown to be substrates for distinct types of carbonyl reductases (for review see [6]).

**Figure 1:**
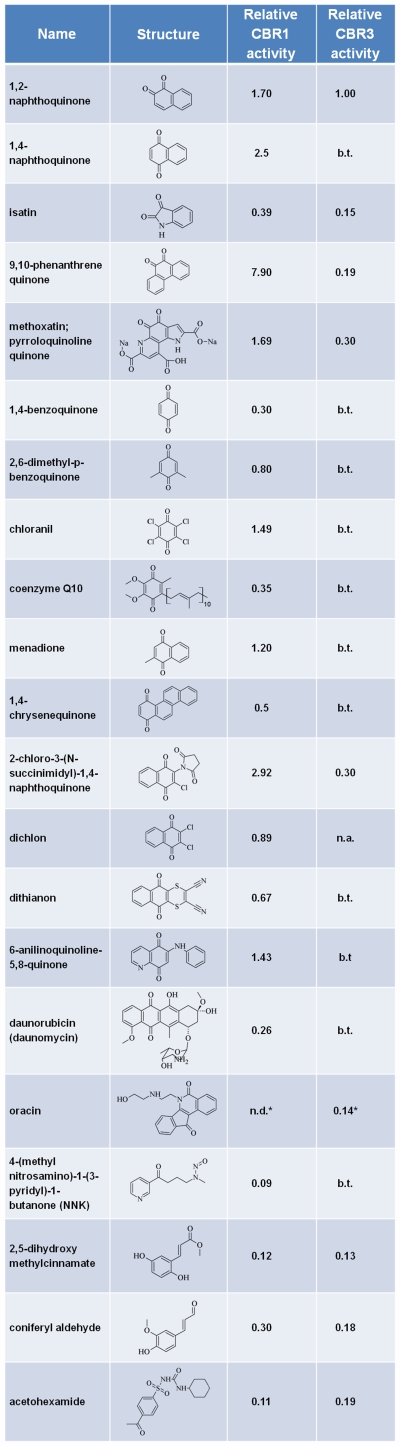
Activity of CBR1 and CBR3 against selected substrates. The threshold for significant activities (measured at 200 µM substrates and 200 nM (∼6.6 µg/ml) enzyme concentration) was set to 10% of the activity of CBR3 against 1,2-naphthoquinone (2.5 µmol/(min mg)), which was set to 1.0 for comparison. All other activities denoted either b.t. (below threshold) or n.a. (no activity observed).

The screening was carried out side-by-side with human CBR1 under identical conditions and revealed that CBR3 has a much narrower substrate spectrum compared to CBR1. Our results confirm the previously recognized broad substrate specificity of CBR1 [1], [3], [6], [9] which is able to metabolize a wide range of substrates including endogenous compounds such as prostaglandins or lipid-derived aldehydes, a wide spectrum of xenobiotics such as ortho- and paraquinones and anthracyclins (supplementary information Table S1). In total, we found significant activity (see below, Figure 1) for 43 out of 111 substrates, with a large fraction of quinones. In contrast, a limited set of substrates were reduced by CBR3 in an NADPH-dependent manner (31 substrates), usually with significantly less activity than CBR1. Among the best substrates for CBR3 was 1,2-naphthoquinone, for which an activity of 2.5 µmol/(min mg) was observed. Compared to the CBR3 activity for 1,2-naphthoquinone a significant (i.e., >10%) activity was observed for 12 out of the 22 tested quinones (Figure 1). Interestingly, a preference of CBR3 for orthoquinones is apparent; no activity was found towards menadione, one of the standard substrates used in activity screens for carbonyl reductases. This is in line with the lack of activity against any other quinone in para configuration. Among the non-quinone compounds that were identified as substrates for CBR3 were isatin and oracin, coniferyl aldehyde and acetohexamide.

We investigated in more detail the difference in activity between CBR1 and CBR3 towards important xenobiotics such as isatin and naphthoquinones (Table 1). The comparison of activities towards isatin and 1,2-naphthoquinone is in line with the observation from the screen: CBR1 showed higher catalytic efficiency for both substrates.

**Table 1:** Comparison of kinetic constants for human CBR1 and CBR3 against selected substrates.

Substrate	CBR1 activity		CBR3 activity	
	Km [µM]	Vmax [µmol/(min mg)]	Km [µM]	Vmax [µmol/(min mg)]
1,2-naphthoquinone	310	11	420	6
1,4-naphthoquinone	560	10	n.a.*	n.a.*
9,10-phenanthrenequinone	35	9	>80**	<0.1**
isatin	2	2	14630	15
oracin	n.d.	n.d.	140	0.1
NNK	7500	3	n.a.*	n.a.*

* very little activity detected.

** no Michaelis-Menten kinetic observed, value estimated from the slope of linear regression of the relation between activity and substrate concentration.

In summary, there are a number of differences between the activity profiles of the two carbonyl reductases. The most striking of them is the strong difference in the activity towards the two naphthoquinones whose only structural difference is the position of the two carbonyl groups (i.e. para vs. ortho). Furthermore, in contrast to CBR1 [9], [11], CBR3 shows no activity towards eicosanoids or aliphatic carbonyls like 4-oxononenal. At this point in time the activity observed against coniferyl aldehyde cannot be assigned to a specific chemical group, i.e. double bond or carbonyl group. Further experiments are required to verify product formation for several of the hits identified.

### Active site architecture of CBR enzymes

To understand the substrate specificity differences between CBR1 and CBR3 we determined the structure of human CBR3 by X-ray crystallography and compared it to recently determined human and porcine structures [12], [20] of CBR1. Based on these structures, residues of potential mechanistic importance were selected for site-directed mutagenetic replacement, and activity of resulting mutants was tested.

**Figure 2:**
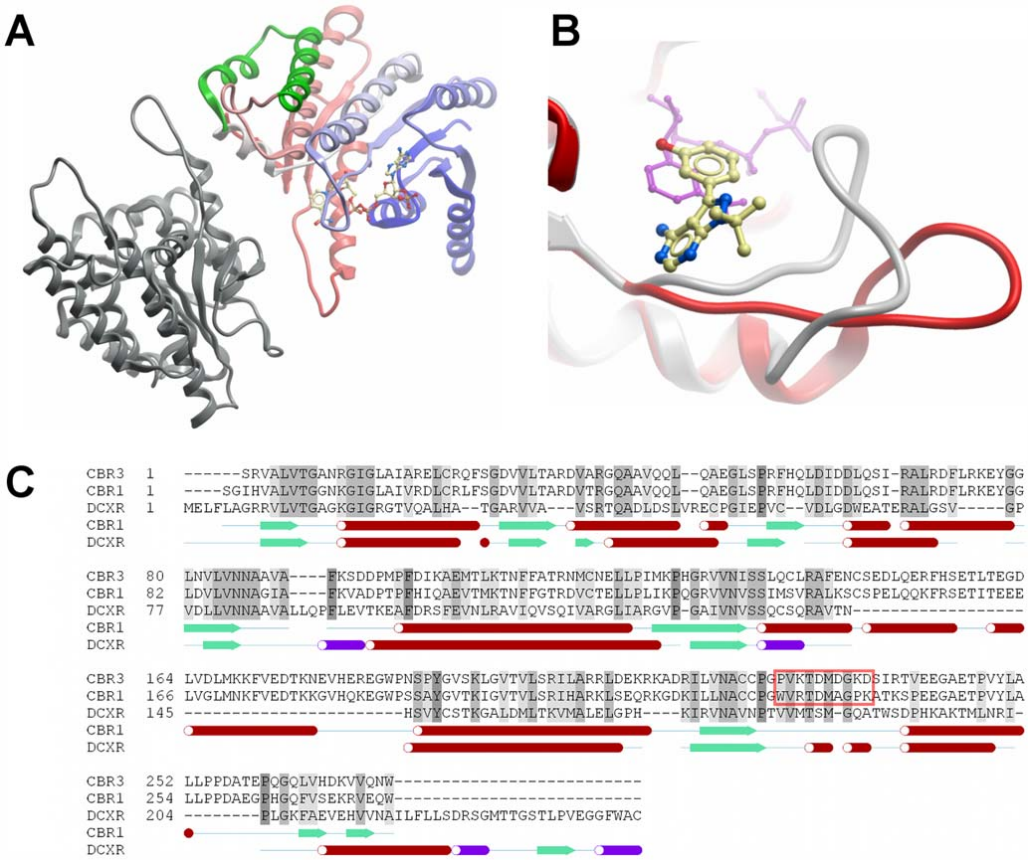
Structure of human CBR3. Panel A: The substrate binding loop in CBR3 is engaged in contacts (red oval) to a symmetry related copy (grey), resulting in an open conformation of the active site. The CBR-specific helical insertion involved in dimerization is highlighted in green. Panel B: Comparison of active site configurations of human CBR enzymes. The overlay of the complex structure of human CBR1 (1wma, in grey) with cofactor (magenta) and inhibitor (ball and stick model) with the binary complex of human CBR3 with NADP (2hrb, in red) shows the open and closed active site loop conformations. Panel C: Sequence alignment of human carbonyl reductases CBR1, CBR3 and dicarbonyl reductase DCXR. The 2-helical insertion found in CBR enzymes is highlighted by green boxing, the active site loop region discussed in this paper is highlighted by a red box. Secondary structure elements are shown for CBR1 and DCXR below the alignment.

The 3D structures of CBR1 and CBR3 are similar, as expected with a canonical Rossmann-fold for nucleotide cofactor binding enzymes of the SDR family [21]. CBRs represent prototypes of monomeric SDRs with a two-helical insertion stabilizing an interface that in other SDRs constitutes the main oligomerization surface (Figure 2).

Inspection of the active sites of the CBR structures reveals an arrangement consistent with the postulated reaction mechanism [21], [22]. Accordingly, Tyr^193/194^ functions as the catalytic acid/base, Ser^139/140^ stabilizes the substrate by forming interactions to the substrate carbonyl, and Lys^197/198^ forms hydrogen bonds with the nicotinamide ribose moiety, thereby lowering the pK_a_ of the Tyr-OH to promote proton transfer. Hydride transfer is from the S-side of C4 of the nicotinamide to the substrate. The role of Asn^113/114^ is to stabilize the position of Lys^197/198^ via a conserved water molecule, and furthermore, to establish a proton relay at the active site, including coenzyme, substrate, Tyr^193/194^, ribose 2'OH, Lys^197/198^, water, and Asn^113/114^.

**Figure 3:**
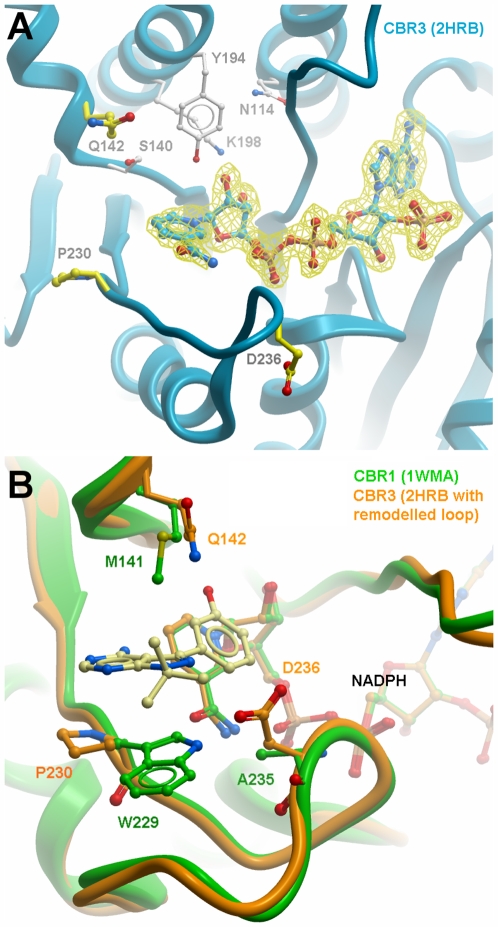
Panel A: Crystal structure of CBR3 (2hrb), close-up of the active site. The cofactor is shown with its electron density map, contoured at 1σ level. Selected residues are shown as sticks. Residues involved in the catalysis are shown with white carbon atoms. Residues used for mutagenesis in this study are shown with yellow carbon atoms. Note that the active site loop is extended, as found in the crystal structure. Panel B: Modelled loop in CBR3 (orange) and comparison to CBR1 (green). Positions and residues used for mutagenesis are shown as sticks. Inhibitor and cofactor from CBR1 (PDB 1wma) are included for reference.

**Figure 4:**
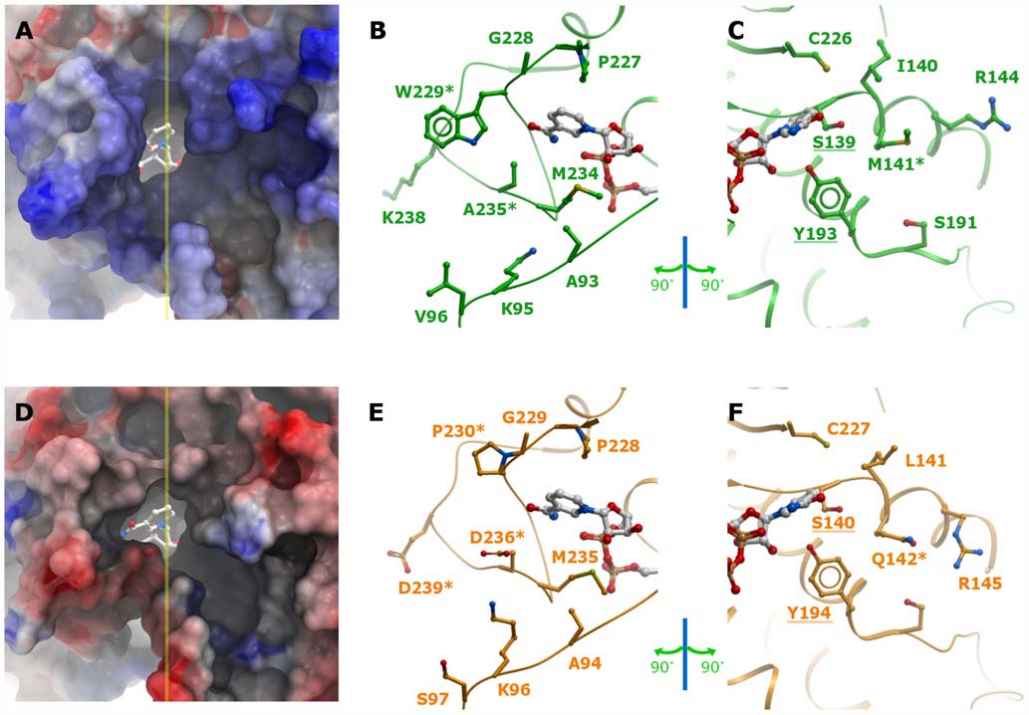
Comparison of active site properties of human CBR1 and CBR3. A–C: CBR1 (green), D–E: CBR3, with modelled loop (orange). First Column (A and D): solvent accessible surface representation of the active site pockets coloured according to electrostatic potentials, with the cofactor represented as sticks. Yellow line marks the plane cutting through the active site. The plane divides the pocket into two halves that are depicted in the following two columns. Second column (B and E): ‘left’ half of the pocket. Third column (C and F): ‘right’ half of the pocket. Cofactor is shown for orientation purpose. Residues that were mutated in this study are marked with asterisks. Catalytic residue labels are underlined.

The main distinguishing feature of the crystal structures of human/porcine CBR1 (ternary cofactor inhibitor complex PDB 1wma [12]; binary cofactor complex PDB 1n5d [20]) and human CBR3 (binary cofactor complex, PDB 2hrb) is the conformation of the substrate binding loop: whereas the CBR1 structures show a conformation with a more closed active site, in CBR3 the loop is engaged in crystal contacts with a symmetry related molecule (Figures 2A and 2B). Despite extensive crystal screening and attempts to obtain ternary complexes, we were unsuccessful in finding different crystal forms. Inspection of the “open” structure reveals that substrate docking in this conformation is not useful to produce models explaining the observed substrate features. We therefore decided to model the CBR3 sequence using the CBR1 structure as template (Figure 3), assuming a similar loop arrangement. The loop modelling results in a conformation with all residues in acceptable regions of a Ramachandran plot, moreover docking analysis with different substrates allowed us to successfully identify critical residues for substrate recognition and catalysis. A comparison of the two CBR structures in the loop-closed conformation shows a wide opening to a gorge-like active site. In the CBR1-inhibitor complex structure (1wma), the inhibitor molecule occupies large parts of the entrance and is also covered by a PEG molecule derived from crystallization. CBR1 has a slightly narrower substrate binding cleft (Figure 4) than CBR3, mainly as a result of the terminal sulf-methyl group of Met^141^. This residue is replaced in CBR3 by Gln^142^ (Figure 4F), which has a similar but not identical conformation, as observed in structures 1wma and 2hrb.

### Critical residues for quinone specificity in CBR enzymes

Comparison of the active sites of CBR1 and CBR3 suggests that three residue positions are critical for substrate recognition and catalysis. In particular, we identified position 229/230 (Trp^229^ in CBR1, Pro^230^ in CBR3), position 235/236 (Ala^235^ in CBR1, Asp^236^ in CBR3) and position 141/142 (Met^141^ in CBR1, Gln^142^ in CBR3) as the most likely candidates for determination of substrate specificity. To analyze the effect of site-directed mutagenetic replacements on activity differences between the two enzymes, we selected isatin and two structural quinone isomers, 1,2- and 1,4-naphthoquinone, as model ortho- and paraquinone substrates.

**Figure 5:**
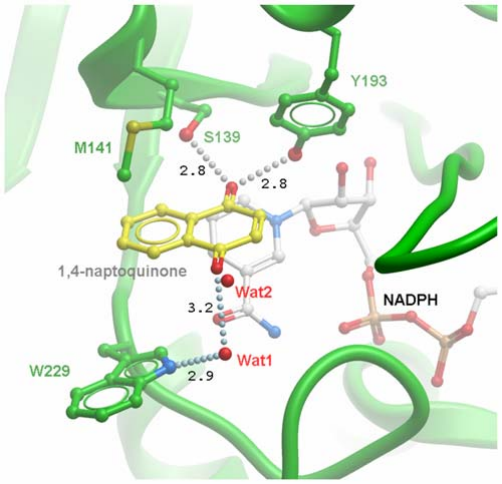
Active site of human CBR1 with 1,4-naphthoquinone docked into a catalytically competent orientation (the water molecules Wat1 and Wat2 were present in the crystal structure of CBR1, but were not used in the docking). The catalytic residues Ser139 and Tyr194 orient the substrate carbonyl, whereas residue Trp229 makes aromatic-stacking interactions and coordination of a water molecule (Wat1) through the indole nitrogen. As a result, the water Wat1 is positioned to form hydrogen bond with the carbonyl group in position to the substrate carbonyl. Note the crystallographic water molecule Wat2 found in the same position as the carbonyl oxygen from the docked substrate. Distances are shown in Å.

A major distinguishing feature between CBR1 and CBR3 is Trp^229^, which is replaced by a prolyl residue in CBR3 (cf Figure 2C). The ternary complex of CBR1 with NADP and OH-PP[12], a high affinity inhibitor, as well as docking studies with different CBR1 substrates suggest a critical role of this residue for substrate selectivity. As deduced from the CBR1-NADP-1,4-naphthoquinone complex model (Figure 5), Trp^229^ serves two possible functions, namely to provide a chemical moiety for aromatic stacking interactions with the substrate, and also to coordinate a water molecule through the indole nitrogen. This water molecule (Wat1), observed in the structure of human CBR1 (PDB 1wma) is putatively responsible for the CBR1 specificity towards para-quinones. This seems to be further reinforced by the position of another water molecule (Wat2) seen in the structure of CBR1, which matches with the C4-carbonyl group of the pose adopted by 1,4-naphthoquinone docked into the active site of CBR1 (Fig. 5). We tested this hypothesis, by replacing Trp^229^ by Pro or Phe, as well as by creating a double mutant Trp^229^Pro/Ala^235^Asp. In CBR1 this second position is located close to Trp^229^ as well as to the nicotinamide and pyrophosphate portions of the cofactor (Figure 3). Both CBR1 Trp^229^ mutants showed significant decrease in activity for 1,4-naphthoquinone and a modest decrease for its ortho- isomer (Table 2). Data for isatin (Table 3) showed drastic increase in K_m_ for Trp^229^Pro mutant while both Trp^229^ substitutions led to faster substrate turnover highlighting the importance of aromatic stacking interactions for substrate recognition and binding. Destabilisation of the active site was much more significant in the Trp^229^Pro/Ala^235^Asp double mutant, where CBR1 residues were exchanged with the corresponding CBR3 residues. It resulted in a 1000-fold increase in K_m_ and a 50-fold increase in V_max_ towards isatin in comparison to WT CBR1 and in a complete loss of activity towards 1,4-naphthoquinone. Both CBR3 Pro^230^ mutants showed some activity towards the para-naphthoquinone but decreased activity for the ortho-naphthoquinone and isatin, as compared with the WT. However, the behaviour of the double Pro^230^Trp/Asp^326^Ala mutant towards naphthoquinones was very similar to the WT CBR3 (Table 2), indicating the possible occurrence of a steric clash between introduced aromatic residue and Asp^236^. In case of isatin, a 2-fold decrease in V_max_ was observed for the Pro^230^Phe mutant, while replacing Asp^236^ with Ala resulted in a significant drop of the K_m_ value, indicating improved binding of the substrate in the active site (Table 3). These data suggest that the residues at both positions are strongly involved in substrate and product binding, indicated by data with swapped residues that either reduce (CBR1) or increase (CBR3) catalytic efficiencies. These residues are part of a more complex set of factors that combine to determine the activity. A major role within this proposed network of interactions falls to Trp^229^ in CBR1.

**Table 2:** Relative catalytic efficiency (k_cat_/K_m_) of CBR1/CBR3 mutants and wild-type proteins against naphthoquinone substrates.

CBR1	1,2-naphthoqui-none [ortho-]	1,4-naphthoqui-none [para-]	CBR3	1,2-naphthoqui-none [ortho-]	1,4-naphthoqui-none [para-]
WT	13.3	6.4	WT	1.0	<0.1
W^229^F	10.4	2.1	P^230^F	0.3	0.3
W^229^P	8.8	0.5	P^230^W	0.2	0.2
W^229^P/A^235^D	1.5	<0.1	P^230^W/D^236^A	0.7	0.1
W^229^P/M^141^A	n.d.*	n.d.*	P^230^W/Q^142^A	0.4	<0.1
W^229^P/M^141^Q	n.d.*	n.d.*	P^230^W/Q^142^M	0.7	<0.1
M^141^Q	9.7	3.5	Q^142^M	1.2	0.3
M^141^A	8.5	1.8	D^236^A	<0.1	0.1

Catalytic efficiency of WT CBR3 against 1,2-naphthoquinone was set to 1.0 for comparison.

* protein unstable.

**Table 3:** Comparison of kinetic constants and relative catalytic efficiency (k_cat_/K_m_) for selected CBR1/CBR3 mutants and wild-type proteins against isatin.

CBR1	K_m_ [µM]	V_max_ [µmol/(mg min)]	Relative activity [1/(µmol min)]	CBR3	K_m_ [µM]	V_max_ [µmol/ (mg min)]	Relative activity [1/(µmol min)]
WT	1.6±0.5	2±0.1	1250	WT	14600±50	14.8±0.1	1
W^229^F	5.1±2.1	10.5±0.7	2060	P^230^F	15500±5000	7.8±2	0.5
W^229^P	40.2±5	19.5±0.8	490	P^230^W	n.t.*	n.t.*	n.t.*
W^229^P/A^235^D	3000±250	120±5	40	P^230^W/D^236^A	5000±400	19.9±1.1	4
				D^236^A	3500±600	37.7±3.6	110

Catalytic efficiency of WT CBR3 was set to 1.0 for comparison.

* not tested.

Residues Met^141^ and Gln^142^, found at homologous positions in CBR1 and CBR3, respectively (Fig. 2C), and displaying similar side-chain conformations are located on helix αF and form the wall opposite of Trp^229^/Pro^230^ (Figure 3B). Replacing this position had a dramatic effect on CBR1: while Met^141^Gln and Met^141^Ala mutants showed modest decrease in activity towards naphthoquinones (Table 2), combining these mutations with Trp^229^Pro led to destabilisation of the protein (very low solubility, no activity). The opposite effect was observed for CBR3, with the Gln^142^Met mutant showing a modest increase in activity, and with the double mutants still active towards 1,2- naphthoquinone.

Taken together, these data highlight the significance of the side-chain chemistry at position 142 in determination of CBR activity.

## Discussion

The objective of this study was to establish a substrate profile for human CBR3, to compare its enzymatic properties to its paralog CBR1, and to establish determinants for activity and substrate specificity. First, CBR3 catalyzes the carbonyl reduction of a much narrower spectrum of xenobiotic substrates in contrast to the exceptionally broad substrate profile of CBR1 [1], [3], [6], [9]. Furthermore, no endogenous substrate could be unequivocally detected to this end for CBR3. Of the known CBR1 substrates, only quinone compounds with ortho substitution or compounds like isatin and the cytostatic oracin could be identified as substrates for human CBR3.

Second, this work establishes the structural basis for narrower substrate specificity in CBR3, and highlights the active site loop found in CBRs as flexible entity that is one critical factor for substrate specificity. Exchange of non-conserved residues between CBR1 and CBR3 in this loop results in position-specific effects that control catalysis. In particular, the data reveal critical roles for Trp^229^ and Pro^230^ in CBR1 and CBR3, respectively, for activity towards para-quinones. These data suggest that hydrophobic interactions as well as possible contacts made through a water molecule coordinated by the indole nitrogen of Trp^229^ could contribute to substrate orientation and possibly product release in the active site. This is supported by the fact, that in the experimental structures, the main-chain of the loop starts to deviate at position 229. This clearly indicates that a major difference between CBR1 and CBR3 is a large, hydrophobic wall built by Trp^229^ in CBR1, and a more open substrate site in CBR3, irrespective if the loop modelling, as performed in this work, is correct or not. Other variable residues found on the loop also contribute to a varying extent to substrate specificity, such as residue Asp^236^ in CBR3, introducing an additional charge into the active site in comparison to CBR1. Other determinants for activity are residues found at position 141/142, namely Met^141^ in CBR1 and Gln^142^ in CBR3. Although of similar size, these residues have significant differential effects on catalytic properties of the active site. A preliminary study [23] showed the whole region encompassing residues 235/236–243/244 as crucial in determination of activities of CBR1 and CBR3. Replacing this region in CBR3 with residues from CBR1, combined with Pro^230^Trp mutation, was sufficient for a 1000-fold increase in activity to ∼40% of activity of CBR1. To summarise, the substrate pockets of CBR1 and CBR3 show fundamental differences in size, as manifested through residues found at position 229, as well as in surface properties, as seen with the more polar residues lining the active site in CBR3 (Figure 4).

Although not specifically tested in this study, it is conceivable that some of the activities observed in this study are related to “propinquity” effects [24]. It has been previously shown that orthoquinones can be reduced to the corresponding hydroquinones in a manner not involving a protein derived catalytic base, as seen with mutant studies performed on members of the aldo-keto reductase [24] or medium-chain dehydrogenase/reductase (MDR) families [25]. Instead, the enzyme is used as a “scaffold” to bring the reduced nicotinamide cofactor and the orthoquinone into close proximity, to allow hydride and direct solvent proton transfer to the adjacent carbonyl groups [24]. This possibility underlines the importance of correct cofactor and substrate positioning in the active site.

As observed from in silico screening (http://www.genecards) and other experimental data [15], CBR1 is an ubiquitously expressed enzyme with highest levels found in liver and the central nervous system, with a significantly lower transcription level but overlapping expression pattern found for CBR3. This suggests some redundancy in substrate specificity, but could also point to different substrates classes and hence different roles for these paralogous enzymes. CBR1 plays without doubt a major role in the phase I metabolism of xenobiotic compounds including xenobiotic quinones [1], [3], [6], [26], a function which we at present cannot wholeheartedly postulate for CBR3, in light of the observed narrow substrate spectrum. In addition, several more recently conducted studies also suggest a critical role for CBR1 in the metabolism of endogenous lipid mediators such as prostaglandins or lipid oxidation products such as the highly reactive and genotoxic 4-oxonon-2-enal, which is produced under oxidative stress [27]–[29]. This could relate the observations that CBR1 is involved in metastasis, neurodegeneration and apoptosis to its properties to catalyze prostaglandin and lipid aldehyde inactivation [9], [11], [27], however, final experimental proof for this hypothesis is necessary. In light of these data, the structural differences determined, and the apparent lack of activity of CBR3 towards lipid mediators like prostaglandins or oxononenal we suggest that CBR3 is likely involved in the metabolism of structurally and chemically different substrates. The precise identity of these compounds needs to be established in further metabolomic and molecular genetic studies.

## Supporting Information

Table S1 Activity screening of human CBR1 and CBR3 against the compounds in the focused carbonyl substrate library. Only activities above 0.25 µmol/(min mg), i.e. above 10% of the activity of CBR3 for 1,2-naphthoquinone, were regarded as significant, otherwise marked as below threshold (b.t.); cases where no activity at all was found are marked ‘n.a.’. Results represent averages ±STDV (n = 3), measured at 200 µM of substrate and 200 nM (∼6.6 µg/ml) of enzyme.
(0.10 MB DOC)

Table S2 Data collection and refinement statistics for human CBR3
(0.03 MB DOC)

Datapack S1 Standalone iSee datapack - contains the enhanced version of this article for use offline. This file can be opened using free software available for download at http://www.molsoft.com/icm_browser.html.
(ICB)

Text S1 Instructions for installation and use of the required web plugin (to access the online enhanced version of this article).
(PDF)
