# Supplementary material for: Structural Basis for Substrate Specificity in Human Monomeric Carbonyl Reductases
Source: PLoS One. 2009 Oct 20;4(10):e7113. doi: 10.1371/journal.pone.0007113 (PMC2741203; doi:10.1371/journal.pone.0007113)
Supplement: Table S1 — Activity screening of human CBR1 and CBR3 against the compounds in the focused carbonyl substrate library. Only activities above 0.25 µmol/(min mg), i.e. above 10% of the activity of CBR3 for 1,2-naphthoquinone, were regarded as significant, otherwise marked as below threshold (b.t.); cases where no activity at all was found are marked ‘n.a.’. Results represent averages ±STDV (n = 3), measured at 200 µM of substrate and 200 nM (∼6.6 µg/ml) of enzyme. (0.10 MB DOC) [file pone.0007113.s001.doc]

**Table S1:** Activity screening of human CBR1 and CBR3 against the compounds in the focused carbonyl substrate library. Only activities above 0.25 μmol/(min mg), i.e. above 10% of the activity of CBR3 for 1,2-naphthoquinone, were regarded as significant, otherwise marked as below threshold (b.t.); cases where no activity at all was found are marked ‘n.a.’. Results represent averages ± STDV (n=3), measured at 200 μM of substrate and 200 nM (~6.6 μg/ml) of enzyme.

| Substrate | CBR1 | CBR3 |
| --- | --- | --- |
| acetaldehyde | b.t. | b.t. |
| acetohexamide | 0.29 ± 0.00 | 0.47 ± 0.17 |
| acetophenone | b.t. | b.t. |
| 4-(1-acetyl-2-oxopropyl)naphthalene-1,2-dione | 13.15 ± 1.42 | 3.20 ± 0.21 |
| acrolein | b.t. | b.t. |
| L-adrenaline | b.t. | 0.29 ± 0.23 |
| adrenosterone | b.t. | b.t. |
| 6-anilinoquinoline-5,8-quinone | 3.57 ± 0.48 | 0.35 ± 0.18 |
| alachlor | 0.26 ± 0.12 | b.t. |
| aldosterone | b.t. | 0.71 ± 0.45 |
| allopregnanolone | b.t. | b.t. |
| androstadienedione | b.t. | b.t. |
| 5alpha-androstane-3beta-ol-16-one | 0.32 ± 0.01 | b.t. |
| 5alpha-androstane-3,11,17-tri-one | 0.56 ± 0.27 | b.t. |
| androstenedione | b.t. | 0.30 ± 0.51 |
| androsterone | b.t. | 0.30 ± 0.03 |
| allopregnane-3,20-dione | b.t. | b.t. |
| benzaldehyde | b.t. | b.t. |
| 1,4-benzoquinone | 13.98 ± 1.50 | 9.66 ± 1.55 |
| benzoylpyridine | 0.90 ± 0.35 | b.t. |
| biochanin A | b.t. | b.t. |
| 2-((3,5-di-tert-butyl-4-hydroxyphenyl)-methylene)-4-cyclopentene-1,3-dione (TX-1123) | b.t. | b.t. |
| chloral | b.t. | b.t. |
| chloranil | 3.72 ± 2.17 | 2.04 ± 0.70 |
| 1,4-chrysenequinone | 0.64 ± 0.26 | b.t. |
| chlordecone | b.t. | b.t. |
| coenzyme Q10 | 0.50 ± 0.45 | b.t. |
| coniferyl aldehyde | 0.92 ± 0.12 | 0.44 ± 0.02 |
| corticosterone | b.t. | b.t. |
| cortisone | b.t. | b.t. |
| cortisol | b.t. | b.t. |
| 1,4-cyclohexanedione | b.t. | n.a. |
| daidzein | 1.19 ± 0.63 | 0.84 ± 1.25 |
| daidzein dimethyl ether | 0.42 ± 0.18 | b.t. |
| daunorubicin | 0.64 ± 0.49 | 0.73 ± 0.01 |
| decanal | b.t. | b.t. |
| dichlon | 2.24 ± 0.01 | b.t. |
| 5alpha-DHT | b.t. | 0.28 ± 0.24 |
| 1,4-dihydroxyanthraquinone | b.t. | b.t. |
| 2,6-dimethyl-p-benzoquinone | 0.78 ± 0.37 | b.t. |
| dithianon | 1.68 ± 0.21 | b.t. |
| doxorubicin | b.t. | b.t. |
| emodin | b.t. | 0.37 ± 0.04 |
| etiocholanone | b.t. | b.t. |
| etiocholane-3beta-ol-17-one | b.t. | b.t. |
| etiocholane-17beta-ol-3-one | 0.34 ± 0.06 | b.t. |
| enoxolone | b.t. | b.t. |
| 2,5-dihydroxymethylcinnamate | 0.31 ± 0.22 | 0.33 ± 0.47 |
| ethacrynic acid | b.t. | b.t. |
| fenofibrate | 0.46 ± 0.27 | 0.26 ± 0.19 |
| genistein | b.t. | 0.37 ± 0.40 |
| DL-glyceraldehyde | b.t. | b.t. |
| 18alpha-glycyrrhetinic acid | b.t. | b.t. |
| haloperidol | 0.33 ± 0.17 | b.t. |
| 2,4-heptadienal | b.t. | b.t. |
| 2,3-heptanedione | 0.27 ± 0.18 | b.t. |
| hexanal | 0.27 ± 0.22 | 0.36 ± 0.26 |
| 2,3,5,6,7,8-hexafluoro-1,4-naphthoquinone | 4.54 ± 1.11 | 0.26 ± 0.25 |
| 3,4-hexanedione | b.t. | b.t. |
| histamine | b.t. | b.t. |
| homogentisic acid | 0.26 ± 0.40 | 0.31 ± 0.33 |
| 21-hydroxyallopregnanolone | 0.42 ± 0.44 | 0.47 ± 0.31 |
| (E)-4-hydroxyhexenal | b.t. | b.t. |
| 2-hydroxy-3-isopropenylcyclohepta-2,4,5-trien-1-one (GK 02122) | 0.27 ± 0.26 | b.t. |
| 4-hydroxynonenal | n.a. | b.t. |
| 17a-hydroxypregnenolone | b.t. | b.t. |
| 17alpha-hydroxyprogesterone | b.t. | b.t. |
| 21-hydroxyprogesterone | b.t. | n.a. |
| isatin | 0.97 ± 0.50 | 0.36 ± 0.23 |
| ketoconazole | 0.44 ± 0.13 | b.t. |
| ketoprofen | b.t. | b.t. |
| malondialdehyde | b.t. | b.t. |
| 2-chloro-3-(N-succinimidyl)-1,4-naphthoquinone | 7.30 ± 1.41 | 0.37 ± 0.25 |
| menadione | 2.04 ± 0.27 | b.t. |
| methoxatin | 4.23 ± 0.93 | 2.21 ± 0.93 |
| mitomycin C | 1.05 ± 0.15 | 0.87 ± 0.34 |
| naltrexone | b.t. | b.t. |
| naloxone | b.t. | 0.27 ± 0.01 |
| 1,2-naphthoquinone | 4.26 ± 0.46 | 2.50 ± 0.59 |
| 1,4-naphthoquinone | 6.26 ± 0.68 | b.t. |
| 4'-nitroacetophenone | b.t. | b.t. |
| NNK | b.t. | b.t. |
| *trans*-2-*cis*-6-nonadienal | b.t. | b.t. |
| γ-nonalactone | 0.70 ± 0.17 | n.a. |
| pentoxifylline | b.t. | b.t. |
| 9,10-phenananthrenquinone | 19.75 ± 4.91 | 0.48 ± 0.34 |
| 1-phenyl-1,2-propanedione | 2.07 ± 0.97 | b.t. |
| phthaldialdehyde | b.t. | b.t. |
| pregnanolone | b.t. | b.t. |
| progesterone | b.t. | b.t. |
| prostaglandin A1 | b.t. | b.t. |
| prostaglandin A2 | b.t. | b.t. |
| prostaglandin E2 | b.t. | b.t. |
| pyridine-2-aldehyde | b.t. | b.t. |
| pyruvaldehyde | 0.46 ± 0.39 | b.t. |
| oracin | n.a. | 0.35 ± 0.10 |
| QC12 | 0.28 ± 0.20 | b.t. |
| testosterone | b.t. | b.t. |
| tetrahydroxy-1,4-quinone | n.a. | b.t. |
| oxysterol 7 keto cholesterol | 1.02 ± 0.22 | 0.63 ± 0.21 |
| raloxifene | b.t. | b.t. |
| all-*trans* retinal | 0.44 ± 0.22 | b.t. |
| serotonin | b.t. | b.t. |
| warfarin | b.t. | b.t. |
