# Supplementary material for: Structural Basis for Substrate Specificity in Human Monomeric Carbonyl Reductases
Source: PLoS One. 2009 Oct 20;4(10):e7113. doi: 10.1371/journal.pone.0007113 (PMC2741203; doi:10.1371/journal.pone.0007113)
Supplement: Table S2 — Data collection and refinement statistics for human CBR3 (0.03 MB DOC) [file pone.0007113.s002.doc]

**Table S2:** Data collection and refinement statistics for human CBR3

| ***PDB code***  ***Data collection***  *X-ray source*  *Wavelength (Å)* | ***2HRB***  *Rigaku FRE-Superbright*  *1.5418* |
| --- | --- |
| Space group | P2_1_2_1_2 |
| Cell dimensions  *a, b, c* (Å)  *α, β, γ* (^o^) | 56.36, 60.14, 88.12  90.0, 90.0, 90.0 |
| Resolution (Å)* | 30.06 – 1.9  (1.95 – 1.9) |
| R_merge_* | 0.093 (0.391) |
| Mean(I/σI)* | 10 (3.0) |
| Redundancy* | 3.4 (3.4) |
| Completeness (%)* | 99.3 (97.9) |
| **Refinement** |  |
| Resolution (Å) | 30.00 – 1.9 |
| No. of reflections | 24 038 |
| *R/R_free_* | 21.5/ 26.4 |
| No. of atoms  Protein  NADP  Glycerol  Water | 2150  48  18  434 |
| R.m.s. deviations  Bond lengths (Å)  Bond angles (^o^) | 0.017  1.646 |

*Highest resolution shell shown in parentheses
